# Serum Prolactin and Macroprolactin Levels among Outpatients with Major Depressive Disorder Following the Administration of Selective Serotonin-Reuptake Inhibitors: A Cross-Sectional Pilot Study

**DOI:** 10.1371/journal.pone.0082749

**Published:** 2013-12-02

**Authors:** Sollip Kim, Young-Min Park

**Affiliations:** 1 Department of Laboratory Medicine, Ilsan Paik Hospital, Inje University College of Medicine, Goyang, Republic of Korea; 2 Department of Psychiatry, Ilsan Paik Hospital, Inje University College of Medicine, Goyang, Republic of Korea; University of Naples Federico II, Italy

## Abstract

Clinical trials evaluating the rate of short-term selective serotonin-reuptake inhibitor (SSRI)-induced hyperprolactinemia have produced conflicting results. Thus, the aim of this study was to clarify whether SSRI therapy can induce hyperprolactinemia and macroprolactinemia. Fifty-five patients with major depressive disorder (MDD) were enrolled in this study. Serum prolactin and macroprolactin levels were measured at a single time point (i.e., in a cross-sectional design). All patients had received SSRI monotherapy (escitalopram, paroxetine, or sertraline) for a mean of 14.75 months. Their mean prolactin level was 15.26 ng/ml. The prevalence of patients with hyperprolactinemia was 10.9% for 6/55, while that of patients with macroprolactinemia was 3.6% for 2/55. The mean prolactin levels were 51.36 and 10.84 ng/ml among those with hyperprolactinemia and a normal prolactin level, respectively. The prolactin level and prevalence of hyperprolactinemia did not differ significantly within each SSRI group. Correlation analysis revealed that there was no correlation between the dosage of each SSRI and prolactin level. These findings suggest that SSRI therapy can induce hyperprolactinemia in patients with MDD. Clinicians should measure and monitor serum prolactin levels, even when both SSRIs and antipsychotics are administered.

## Introduction

There is evidence that selective serotonin-reuptake inhibitors (SSRIs) can induce hyperprolactinemia, which causes galactorrhea, gynecomastia, hormonal abnormality, and sexual dysfunction [1,2]. Even Cohen and Davies reported that SSRIs were most frequent cause of drug-induced hyperprolactinemia [3]. However, most of clinical trials designed to evaluate prospectively the rate of short-term (1 day-12 weeks) SSRI-induced hyperprolactinemia have produced conflicting results [4], with some studies showing that SSRIs can increase the level of serum prolactin (PRL) [5–8], while others demonstrating no such increase [9–13]. Papakostas and colleagues recently reported that 4.5% of men and 22.2% of women with major depressive disorder (MDD) developed new-onset hyperprolactinemia following treatment with fluoxetine [14]. Macroprolactin essentially comprises **a** complex of PRL with immunoglobulin G (IgG), especially anti-PRL autoantibodies [15]. In addition, macroprolactinemia is a heterogeneous state with various causes, with 87% of macroprolactin comprising PRL-IgG complex and 67% being autoantibody-bound PRL [15,16]. However, macroprolactin does not seem to induce hyperprolactinemia-related adverse effects due to its low bioactivity. 

Most of these previous prolactin studies analyzed the condition of patients with short-duration SSRI therapy. The purpose of this study was to clarify whether relatively long-term SSRI therapy can induce hyperprolactinemia. Both PRL and macroprolactin levels were measured, the latter because it is clinically important in that it has low bioactivity. To our knowledge this is the first study to examine macroprolactin levels in patients with MDD who received SSRI therapy.

## Materials

### Subjects

In total, 55 outpatients who met the criteria in the Diagnostic and Statistical Manual of Mental Disorders, 4th edition (DSM-IV) for MDD were enrolled in this study. The subjects, who had been receiving long-term (mean, 14.75 months) SSRI medication, were recruited from an outpatient clinic. They had not been diagnosed with any major mental disorders on axis I or axis II of the DSM-IV (including schizophrenia, bipolar disorder, anxiety disorder, eating disorder, or borderline personality disorder) or major medical and/or neurological disorders. Patients taking psychotropic agents other than hypnotic drugs (benzodiazepine or zolpidem) and SSRIs including escitalopram, paroxetine, or sertraline were excluded. The study was conducted from January 2012 to April 2013. Written informed consent to participate was obtained from all patients before beginning the investigation, and the applied protocol was approved by the institutional review board of Inje University.

### Study design

Serum PRL and macroprolactin levels were measured at a single time point (i.e., in a cross-sectional design). All patients had received SSRI monotherapy including escitalopram (*n*=35), paroxetine (*n*=24), or sertraline (*n*=6) for a mean of 14.75 (range, 2–84) months. A blood sample was taken from each subject in the morning in a fasting state (91%, 50/55). The normal range of PRL levels reported in the hospital laboratory is 4.0–15.2 ng/ml in men and 4.8–23.3 ng/ml in women.

### Measurements of PRL and macroprolactin

Macroprolactinemia screening was performed as described previously [17]. Serum samples were precipitated with polyethylene glycol (PEG; PEG 8000, Sigma-Aldrich, St. Louis, MO, USA) and the supernatants were assayed for PRL using Roche PRL assays (Roche Diagnostics, Indianapolis, IN, USA). The results were compared with those of direct serum PRL assays. The PRL concentration in the supernatant after PEG treatment was defined as free PRL, and the concentration without PEG treatment was defined as total PRL. The PEG-precipitated PRL was calculated as a percentage using the following equation: (total PRL–free PRL)/total PRL×100. A value greater than 52.8% [corresponding to the mean plus two standard deviations (SDs)] defined macroprolactinemia in this study. The concentration of PRL was measured using an automated chemiluminescence assay (Roche Diagnostics).

### Analysis

All statistical analyses were carried out using the SAS 9.3 software package and SALT 2.5, and all results are reported as mean±SD values. The demographic and clinical variables, PRL levels, and frequencies of some group variables were compared using Student’s *t* test, ANOVA, the chi-square test, and correlation analysis (Pearson’s correlation). Student’s *t* test was used to determine the mean and SD values, and whether there were significant differences between groups with respect to age, PRL level, macroprolactin level, and treatment duration of SSRIs. ANOVA and the Tukey post-hoc test were used to determine the PRL and macroprolactin levels relative to each SSRI, and the chi-square test was used to determine whether the difference among groups was significant with respect to the frequency of hyperprolactinemia according to each antidepressant. Correlation analysis using Pearson’s correlation was carried out to establish clearly whether there was any significant correlation between the treatment duration of each SSRI and PRL level. All tests were two tailed, and group differences were considered to be significant when p<0.05.

## Results

The mean PRL level before PEG and the free PRL level after PEG in our sample of 55 patients with MDD were 15.26 and 11.29 ng/ml, respectively, and the mean SSRI treatment duration for the current episode was 14.75 months. The prevalence rates of patients with hyperprolactinemia and macroprolactinemia were 10.9% for 6/55 and 3.6% for 2/55, respectively (Table 1). The PRL level did not differ significantly between males (14.41±8.74 ng/ml) and females (15.52±24.24 ng/ml, *p*=0.87) (Table 2). Two male and four female patients presented with hyperprolactinemia. However, none of the patients with hyperprolactinemia also had macroprolactinemia; all of the patients with macroprolactinemia (*n*=2) had PRL levels within the normal range, even before PEG treatment (Table 1). The PRL level did not differ between patients who had received SSRI monotherapy for at least 6 months (*n*=34) and for less than 6 months (*n*=21), respectively. 

**Table 1:** Demographic and clinical variables in normal prolactin group and hyperprolactinemia group.

Variables	Normal PRL group (n=49)	Hyperprolactinemia group (n=6)	p-value
Age (years)	48.43±17.19	45.0±23.26	0.66
Sex (M/F)*	11/38	2/4	0.62
Treatment duration (months)	14.29±18.41	18.83±16.56	0.57
HAMD	6.27±5.33	9.33±6.09	0.19
PRL level before PEG (ng/ml)	10.84±4.71	51.36±55.08	0.0000019**
PRL level after PEG (ng/ml)	8.47±3.80	34.35±28.05	0.000000041**
PEG-precipitated PRL (%)	23.99±11.93	24.44±9.81	0.93
Macroprolactinemia (yes/no)	2/47	0/6	N/A

* Fisher's Exact Test, ** P<0.05, PRL; prolactin, N/A; nonavailable

**Table 2:** Demographic and clinical variables between male and female groups.

Variables	Male (n=13)	Female (n=42)	p-value
Age (years)	41.38±18.21	50.12±17.26	0.12
Treatment duration (months)	8.31±8.98	16.79±19.80	0.14
PRL level before PEG (ng/ml)	14.41±8.73	15.52±24.23	0.87
PRL level after PEG (ng/ml)	10.87±7.12	11.42±13.61	0.89
PEG-precipitated PRL (%)	25.33±11.26	23.64±11.86	0.65
Macroprolactinemia (yes/no)*	1/13	1/42	0.42

* Fisher's Exact Test, PRL; prolactin

**Figure 1:**
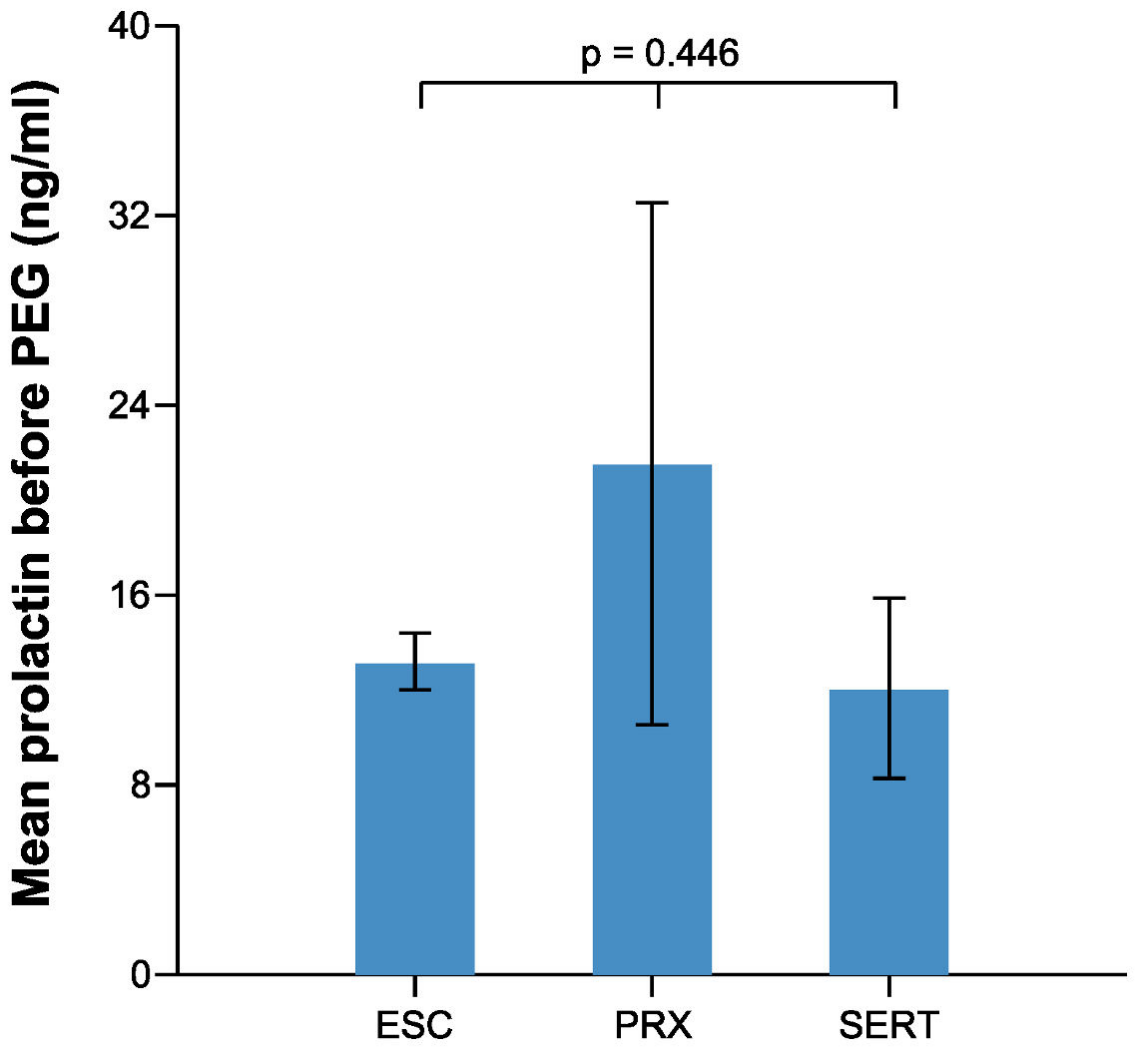
Mean prolactin levels before PEG among patients taking escitalopram, paroxetine, and sertraline (ESC; escitalopram, PRX; paroxetine, SERT; sertraline).

**Figure 2:**
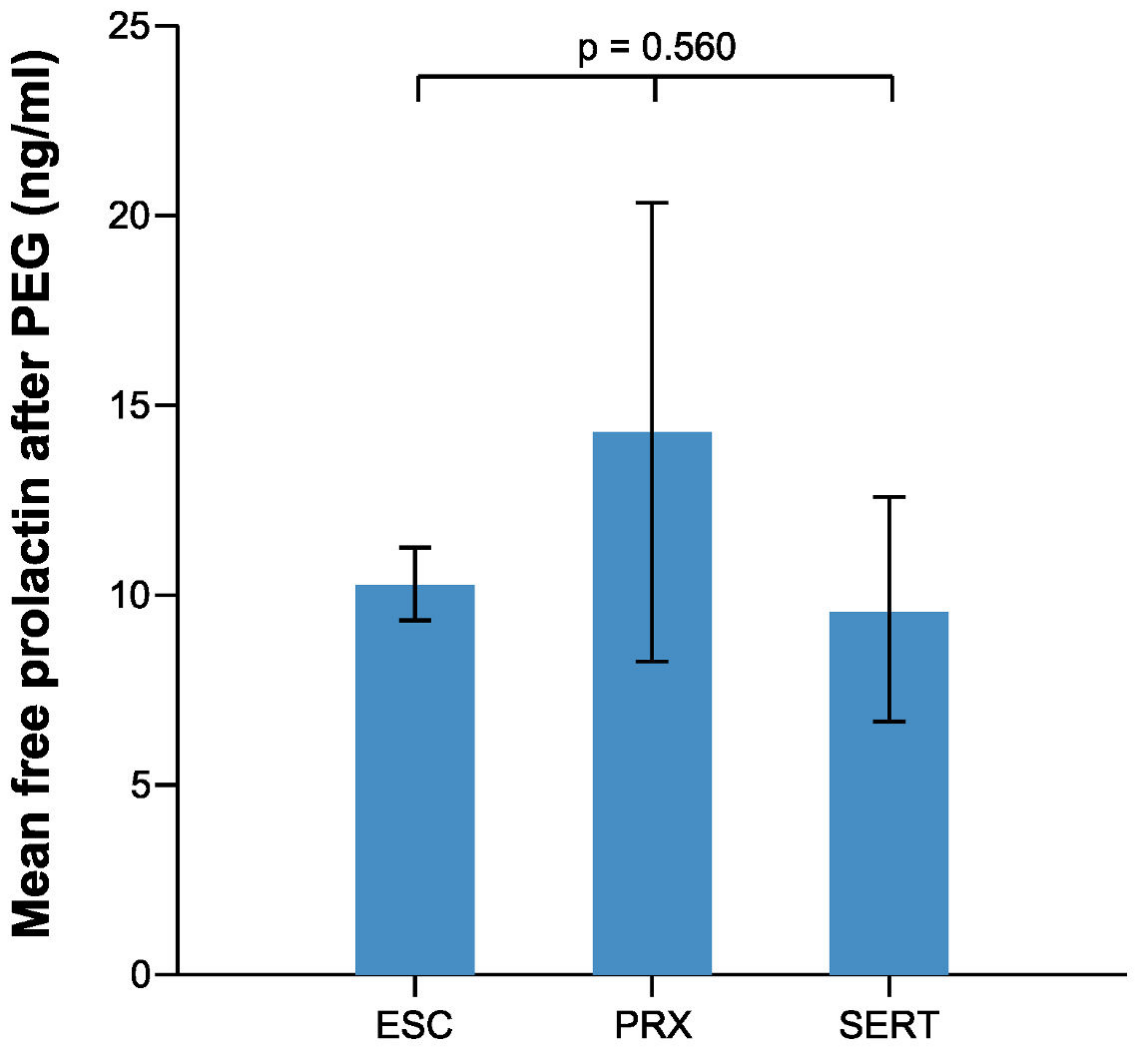
Mean prolactin levels after PEG among patients taking escitalopram, paroxetine, and sertraline (ESC; escitalopram, PRX; paroxetine, SERT; sertraline).

**Figure 3:**
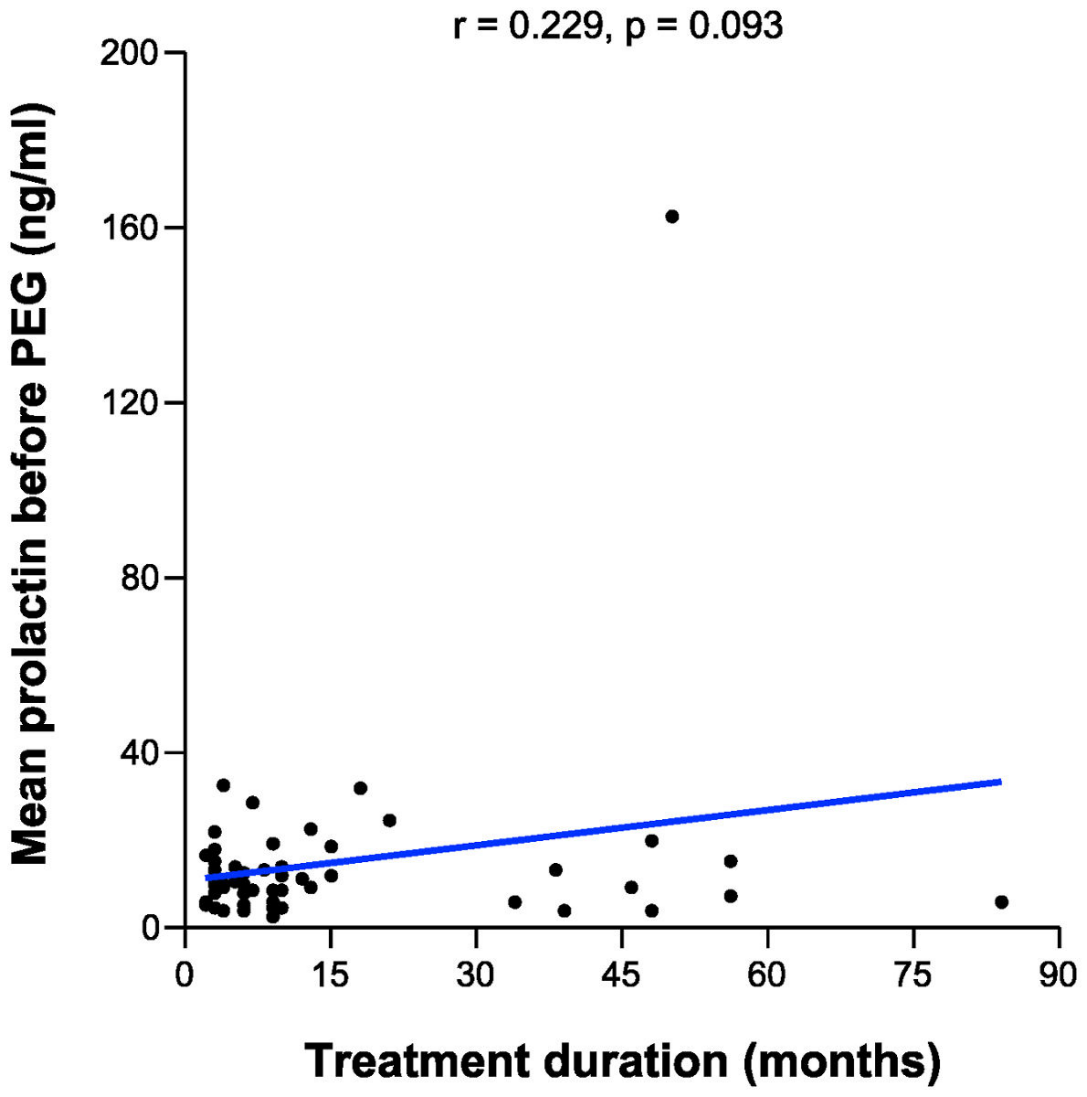
Correlation between the treatment duration of each SSRI and prolactin levels.

**Figure 4:**
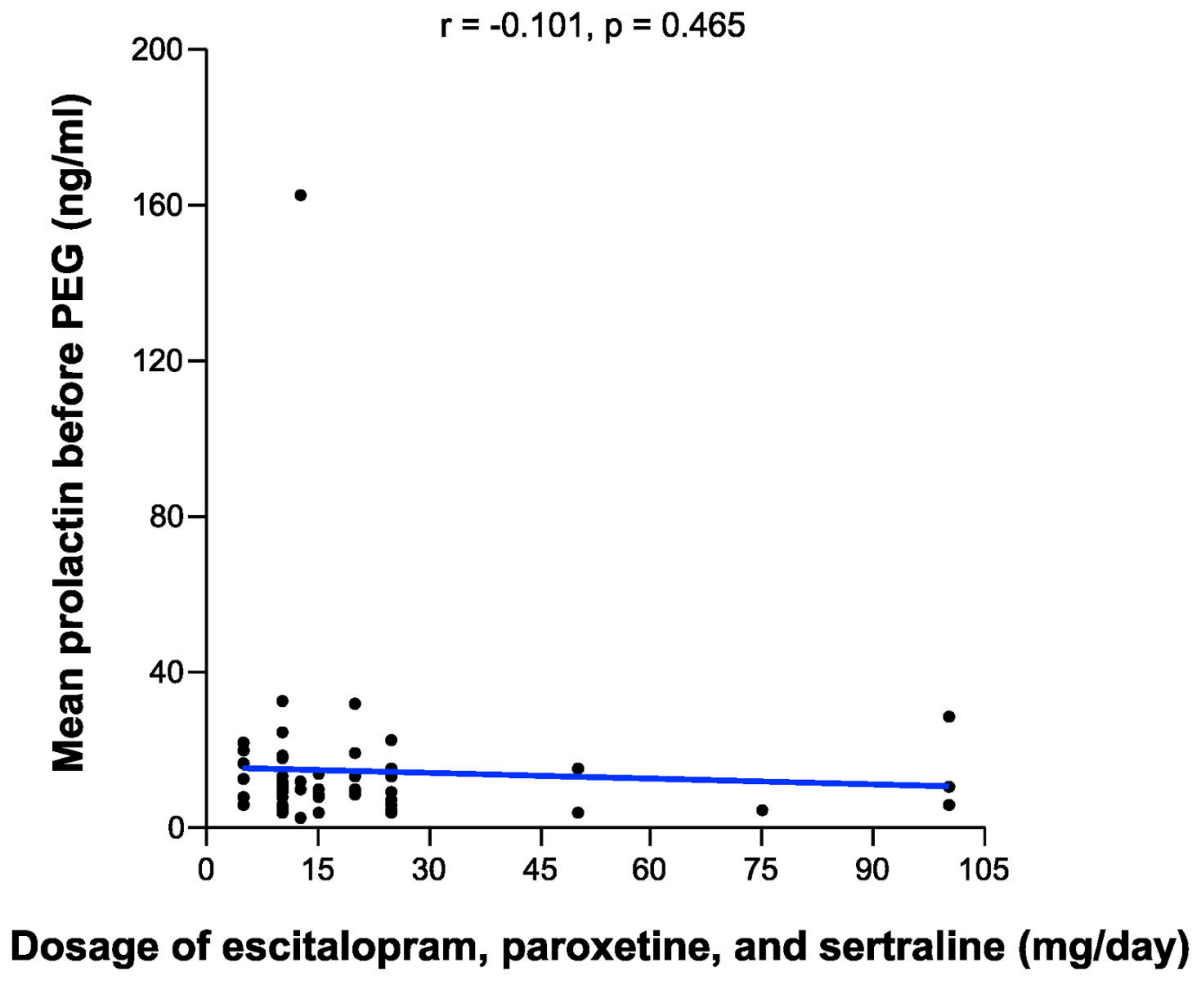
Correlation between the dosage of each SSRI and prolactin levels.

The mean PRL levels were 51.36 and 10.84 ng/ml among those with hyperprolactinemia and normal PRL levels, respectively (Table 1). The mean PRL levels before PEG treatment among those taking escitalopram, paroxetine, and sertraline were 13.25, 21.62, and 12.15 ng/ml, respectively; the mean free prolactin levels after PEG treatment were 10.34, 14.37, and 9.64 ng/ml, respectively (Table 3). There was no statistically significant difference among each SSRI group with respect to mean PRL level before PEG treatment or mean free PRL level after PEG treatment (Figure 1, 2, and Table 3). In addition, there was no statistically significant difference between pairs of SSRI groups in prolactin levels (escitalopram vs paroxetine, paroxetine vs sertraline, and escitalopram vs sertraline) (Tukey post-hoc analysis, p>0.05). The frequency of patients with hyperprolactinemia also did not differ within each SSRI group (Table 3). Correlation analysis revealed no correlation between the treatment duration or dosage of each SSRI and PRL level (Figure 3, Figure 4).

**Table 3:** Comparison of prolactin levels and the frequency of subjects with hyperprolactinemia among each SSRI group.

Variables	ESC	PRX	SERT	p-value
PRL level before PEG (ng/ml)	13.25±7.23	21.62±41.17	12.15±9.29	0.45
PRL level after PEG (ng/ml)	10.34±5.75	14.37±22.60	9.64±7.25	0.56
Hyperprolactinemia (yes/no)*	3/32	2/12	1/5	0.53

PRL; prolactin, ESC;escitalopram, PRX;paroxetine, SERT; sertraline,

* Fisher's Exact Test

## Discussion

The results presented herein revealed that the prevalence of SSRI-induced hyperprolactinemia was 10.9%. This value is similar to that of 12.5% found by Papakostas and colleagues in a cohort with hyperprolactinemia induced following 12 weeks of fluoxetine [14]. In addition, Pearson’s correlation revealed no significant correlation between the SSRI treatment duration and PRL level in the present study (*r*=0.229, *p*=0.093). Thus, it seems that the treatment duration of SSRIs does not have a major impact on hyperprolactinemia.

Some studies, including the present one, have shown that SSRIs can increase the serum level of PRL [5–8], and yet others have found no such increase in serum PRL [9–13]. Thus, the findings of SSRI-induced hyperprolactinemia remain a matter of controversy. However, the mean PRL level was 28.93 ng/ml for five of the six patients with hyperprolactinemia in the present study; the sixth one had marked hyperprolactinemia (163.5 ng/ml). In addition, the mean PRL level did not differ between males and females. These mean that the usual SSRI therapy can increase PRL levels, but that their impact on PRL level is not necessarily strong. Coker and Taylor also advocated that prolactin-related effects of antidepressants are difficult to predict but that symptomatic events are likely to be very infrequent, and hence they suggested that routine screening test of prolactin level is not required [18].

Macroprolactinemia is defined as a condition in which macroprolactin—which has a molecular mass of >150 kDa and, unlike monomeric PRL, has no action in the human body—is predominantly present in the serum [a PEG-precipitated PRL level greater than 52.8% (mean+2SD) was defined as macroprolactinemia in this study] [15,19]. The prevalence of macroprolactinemia has been estimated at 10–46% in patients with hyperprolactinemia [20–26]. However, none of the patients with hyperprolactinemia in the present study exhibited macroprolactinemia. While one of the patients in the present study exhibited a markedly elevated PRL level of 163.5 ng/ml, her PEG-precipitated PRL (44.15%) was the highest of all of the patients with hyperprolactinemia. However, two patients with macroprolactinemia had no hyperprolactinemia; their PRL levels were 12.04 and 15.85 ng/ml. This contrasts with reports that hyperprolactinemia often occurs in patients with macroprolactinemia because of the delayed clearance of macroprolactin [15,27]. This prompted some investigators to suggest that clinicians should screen for macroprolactinemia in all patients with hyperprolactinemia in order to avoid unnecessary examinations and treatments [15].

There was no significant difference within each SSRI group with respect to the mean PRL level before and after PEG treatment and the mean free prolactin level after PEG treatment (Figure 1, 2). In addition, there was no difference in the frequency of patients with hyperprolactinemia. One study showed that the mean PRL level was lower in patients treated with sertraline than in those treated with other SSRIs [11]. An explanation for this finding is that sertraline is a weak dopamine-reuptake inhibitor, a characteristic that could counteract the action of serotonin [28,29]. Although the number of patients taking sertraline was small in the present study (n=6), that finding was not corroborated herein. 

Correlation analysis revealed no correlation between the dosage of each SSRI and PRL level. This result differs from that for antipsychotic-induced hyperprolactinemia: most antipsychotic-induced hyperprolactinemia studies have found a positive correlation between antipsychotic dosage and PRL level [30–33]. 

This study was subject to several limitations. Its cross-sectional design meant that baseline PRL levels were not measured, making it impossible to differentiate a cause-and-effect relationship from a simple association. In addition, the sample was relatively small due to the one-center design of this study. However, this is a relatively long-term study related to SSRI-induced hyperprolactinemia, since the mean treatment duration of SSRIs was 14.75 months, in contrast to the previous, shorter-term studies (range, 1 day-12 weeks). In addition, this is the first study to examine macroprolactin levels in patients with MDD who received SSRI therapy. Furthermore, all of the patients are Korean and outpatients with MDD, and so the sample was relatively homogeneous. 

The present findings suggest that SSRI therapy can induce hyperprolactinemia in patients with MDD. Clinicians should measure and monitor serum PRL levels in these patients, even when they are receiving both SSRIs and antipsychotics. 
